# Full normalization of severe hypertension after parathryoidectomy – a case report and systematic review

**DOI:** 10.1186/s12882-018-0900-y

**Published:** 2018-05-11

**Authors:** Andreea Corina Sofronie, Isabelle Kooij, Claude Bursot, Giulia Santagati, Jean-Philippe Coindre, Giorgina Barbara Piccoli

**Affiliations:** 1 Nephrology Centre Hospitalier Le Mans, Le Mans, France; 2 Dipartimento di Scienze Cliniche e Biologiche, Università di Torino, Turin, Italy; 3 Nuclear Medicine, Centre Hospitalier Le Mans, Le Mans, France; 4 Nephrology Centre Hospitalier Le Mans, 194 Avenue Rubillard 72000, Le Mans, France

**Keywords:** Parathyroidectomy, Dialysis, Hypertension, Case report, Systematic review

## Abstract

**Background:**

Although the relationship between hyperparathyroidism and hypertension has been described for decades, the role of hyperparathyroidism in hypertension in dialysis is still unclear. Following the case of a severely hypertensive dialysis patient, in which parathyroidectomy (PTX) corrected the metabolic imbalance and normalized blood pressure (BP), we tried to contextualize our observation with a systematic review of the recent literature on the effect of PTX on BP.

**Case presentation:**

A dialysis patient, aged 19 years at the time of this report, with chronic kidney disease (CKD) from childhood; he was an early-preterm baby with very low birth weight (910 g), and is affected by a so-far unidentified familial nephropathy. He started dialysis in emergency at the age of 17. Except for low-dose Bisoprolol, he refused all chronic medication; hypertension (165–200/90–130 mmHg) did not respond to attainment of dry weight (Kt/V > 1.7; BNP 70–200 pg/ml pre-dialysis). He underwent subtotal PTX 1 year after dialysis start; after PTX, his blood pressure stabilized in the 100–140/50–80 range, and is normal without treatment 5 months later.

**Conclusion:**

Our patient has some peculiar features: he is young, has a non-immunologic disease, poor compliance to drug therapy, excellent dialysis efficiency. His lack of compliance allows observing the effect of PTX on BP without pharmacologic interference.

The prompt, complete and long-lasting BP normalization led us to systematic review the current literature (Pubmed, Embase, Cochrane Collaboration 2000–2016) retrieving 8 case series (194 cases), and one case report (3 patients).

The meta-analysis showed a significant, albeit moderate, improvement in BP after PTX (difference: systolic BP -8.49 (CI 2.21–14.58) mmHg; diastolic BP -4.14 (CI 1.45–6.84) mmHg); analysis is not fully conclusive due to lack of information on anti-hypertensive agents. The 3 cases reported displayed a sharp reduction in BP after PTX.

In summary, PTX may have a positive influence on BP control, and may result in complete correction or even hypotension in some patients. The potential clinical relevance of this relationship warrants prospective large-scale studies.

## Background

Hyperparathyroidism has long been associated with arterial hypertension [1, 2]. The mechanisms at the basis of this effect are only partially understood. They are probably modulated by a series of factors, including the severity of the hyperparathyroidism, its type and the presence of other metabolic derangements. While parathyroid hormone (PTH) itself is a vasodilator, perhaps as the result of increased prostaglandin production, hyperparathyroidism in end stage renal disease is thought to produce hypertension by encouraging entry of calcium into the smooth muscle cells vascular walls [3–6].

If excess PTH induces hypertension, a decline in blood pressure should follow ablation of parathyroid function in dialysis patients with severe hyperparathyroidism and hypertension. Overall, the data suggest that, at least in selected patients, parathyroidectomy (PTX) is associated with an improvement in blood pressure control [7–13]. However, the entity of the effect is not entirely clear, as well as the interference between blood calcium levels, dialysate calcium and the overall intradialytic calcium mass balance [7, 14, 15].

The practice of PTX in dialysis patients has changed over time. While in the past surgery often represented the only way to correct a severe metabolic imbalance in the context of hyperparathyroidism, improvements in dialysis efficacy, and the new generations of drugs active on the Ca-Phosphate-PTH axis have sharply reduced the need for PTX. The issue is however still open, and in recent years a number of large studies have suggested reconsidering PTX, not only for the immediate metabolic advantages it produces, but also because of an apparent survival advantage in patients who underwent surgical correction [16–23].

In this context, several recent studies addressed the relationship between cardiovascular diseases and PTX. While a positive effect on overall cardiovascular status is reported in many large series, some papers considered the unsolved issue of the relationship between hyperparathyroidism and elevated blood pressure in dialysis patients, once more leading to controversial results [9, 12, 13, 17].

It is in this context that we would like to report on an emblematic case, a young dialysis patient whose severe hypertension was fully corrected by PTX, and review the recent literature on this subject, to underline the importance of this intriguing relationship, which is rarely adequately taken into account in current clinical practice.

## Case presentation

A young dialysis patient, aged 19 years at the time of the present report. Chronic kidney disease (CKD) was evident from childhood: at 9 years of age his serum creatinine was 1.04 mg/dL (92 μmol/L). The patient was an early-preterm baby (born at 30 weeks) with very low birth weight (910 g), both elements associated with an increased risk of developing several metabolic diseases including CKD [24].

He is affected by a so far unidentified familial kidney disease: his 57-year-old mother has Stage 4 CKD and his sister underwent preemptive kidney transplantation at the age of 22. In all family members CKD is characterized by low-grade proteinuria, relatively high uric acid levels, and no microhematuria, consistent with the hypothesis of an interstitial nephropathy. The family history is marked by low compliance with treatments and controls, and a precise characterization of the genetic disease has not yet been possible. A search for mutations of UMOD and REN, and the enzyme assessment for Fabry’s disease, tested negative; HLA-B27 is negative; his karyotype is normal and no other clinical element suggested other common monogenic nephropathy (no deafness, visual disturbances, angiokeratoma). Genetic reevaluation is on-going.

The patient started dialysis in emergency at the age of 17, in the presence of extreme uremia and generalized edema. At referral, his serum creatinine was 18.43 mg/dl (1630 umol/l), urea 524.32 mg/dl (87.3 mmol/l), bicarbonate 13 mmol/l, potassium 3.8 mmol/l, hemoglobin level 6.1 g/dl, BNP > 4000 pg/ml. In keeping with a chronic, non-compensated end-stage kidney disease, PTH was extremely elevated (> 1500 ng/l), with normal calcium and high phosphate levels (Table 1).

**Table 1:** Clinical and laboratory characteristics of our patient before and after parathyroidectomy

Start of dialysis (12/2015)	No medication Start of dialysis in emergency ᅟ Biochemical data: BUN: 245 mg/dl (40.8 mmol/l), creatinine: 18.43 mg/dl (1630 umol/l), bicarbonates: 13 mmol/l, albumin: 2.6 g/dl; BNP: > 4000 pg/ml. PTH: > 1500 ng/l; Calcium: 8.52 mg/dl (2.13 mmol/l); Phosphate: 10.49 mg/dl (3.39 mmol/l) ᅟ Predialysis BP: 165–200 mmHg (systolic), 90–130 mmHg (diastolic)
Pre-parathyroidectomy (10/2016)	Medication: Bisoprolol 2.5 mg Dialysis schedule: 4 h × 3 weekly sessions; access: native AV fistula; Bicarbonate buffer, Ca 1.5 mmol/l, Na 138 mEq/l Biochemical data: BUN: 41.22 mg/dl (14.7 mmol/l), creatinine: 12.85 mg/dl (1136 umol/l), bicarbonates: 19 mmol/l, BNP: > 1500 pg/ml, Calcium: 10.12 mg/dl (2.53 mmol/l), PTH: 1317 ng/l, ionized Calcium: 5.04 mg/dl (1.26 mmol/l), phosphorus: 7.58 mg/dl (2.45 mmol/l), albumin: 3.5 g/dl, vitamin D 25 OH: 39 μg/l ᅟ Kt/V (Daugirdas 2): 1.7; nPCR: 1.2 g/kg/day ᅟ Predialysis BP: 150–180 (systolic), 90–100 mmHg (diastolic)
First week post parathyroidectomy (11/2016)	Medication: Bisoprolol 2.5 mg, Calcium carbonate 1.54 g (3 times per day), Alfacalcidol 0.5 μg Dialysis schedule: 4 h × 3 weekly sessions; access: native AV fistula; Bicarbonate buffer, Ca 1.5 mmol/l Na 138 mEq/l ᅟ Biochemical data: BUN: 54.34 mg/dl (9.06 mmol/l), creatinine: 11.48 mg/dl (1015 umol/l), bicarbonates: 19 mmol/l, PTH: 70 ng/l, Calcium: 7.12 mg/dl (1.78 mmol/l); Ionised Calcium: 3.48 mg/dl (0.87 mmol/l), albumin: 3.3 g/dl ᅟ Predialisys BP: 120–135 (systolic), 60–80 mmHg (diastolic)
Four months post parathyroidectomy (02/2017)	Medication: Bisoprolol 2.5 mg, Cholecalciferol 100,000 UI (1 vial per month), Calcium carbonate 1.54 g (2 times per day), Alfacalcidol 1 μg ᅟ Dialysis schedule: 4 h × 3 weekly sessions; access: native AV fistula; Bicarbonate buffer, Ca 1.5 mmol/l, Na 138 mEq/l ᅟ Biochemical data: BUN: 34.39 mg/dl (12.28 mmol/l), creatinine = 12.23 mg/dl (1082 umol/l), bicarbonates: 15 mmol/l, PTH: 199 ng/l, Calcium: 9.2 mg/dl (2.3 mmol/l); Ionised Calcium: 4.84 mg/dl (1.21 mmol/l), albumin: 3.6 g/dl ᅟ Predialysis arterial pressure 120–130 (systolic); 70–85 mmHg (diastolic) ᅟ Kt/V (Daugirdas 2) 1.7; nPCR: 1.2 g/kg/day

*BNP* B-type natriuretic peptide, *BP* blood pressure, *BUN* blood urea nitrogen, *nPCR* protein catabolic rate, *PTH* parathyroid hormone

With the start of hemodialysis, he became severely hypertensive and blood pressure did not respond to weight loss. Except for low-dose Bisoprolol, started 6 months previously to prevent the subjective symptom of “palpitations” at the end of dialysis (corresponding to a heath rate of 80–100/min at the end of the treatment), which we hoped could at least partially ameliorate his severe hypertension, the patient took no chronic medications.

His blood pressure did not decrease during dialysis, although he had no clinical sign of chronic overload, no radiological sign of pulmonary overload, no oedema and normal BNP levels (between 70 and 200 pg/ml pre-dialysis). His usual weight gain between sessions was 1.5–2.5 Kg, and did not change before and after parathyroidectomy.

At time of dialysis start, his echocardiography showed non obstructive homogenous left ventricular hypertrophy with an ejection fraction of 35–40% and small circumferential pericardial effusion. The ejection fraction was restored 1 year after dialysis start (50%), shortly before the parathyroidectomy and the left ventricular hypertrophy regressed (116 g/m2 versus 172 g/m2). Due to the essential normalisation of the picture, a further control was not performed after parathyroidectomy, and is scheduled 2 years after the previous one.

All viral and immunologic tests (VHB, VHC, HIV-1 and 2, HTLV-1 and 2, antiphospholipid, antinuclear, anti DNA, anti SSA, anti-SSB, anti-Sm, anti-RNP, anti-histones, anti-PM/SCL, anti-PCNA, anti-Scl 70, anti-JO-1 antibodies, anticentromere, anti-endomisium, anti-mitochondrial type 2 and 5, anti-gp210, anti Sp100, anti SLA, anti-LKM, anti-actin antibodies) tested negative at referral (with the exception of smooth muscle antibodies at low titer: 1/200). The results were confirmed at a further recent control.

His predialysis blood pressure range, in the last month before parathyroidectomy was 165–200/90–130 mmHg, while post dialysis it was 170–190/80–100 mmHg.

His usual weight gain between sessions was 1.5–2.5 Kg, and did not change before and after parathyroidectomy.

Lack of compliance with the chronic treatment was not accompanied by lack of compliance with dialysis. In fact, he never missed a dialysis session, nor did he ever shorten dialysis time. It should be noted that his dialysis efficiency was over target, and his nutritional status was good. His chronic dialysis data are summarized in Table 1.

**Fig. 1:**
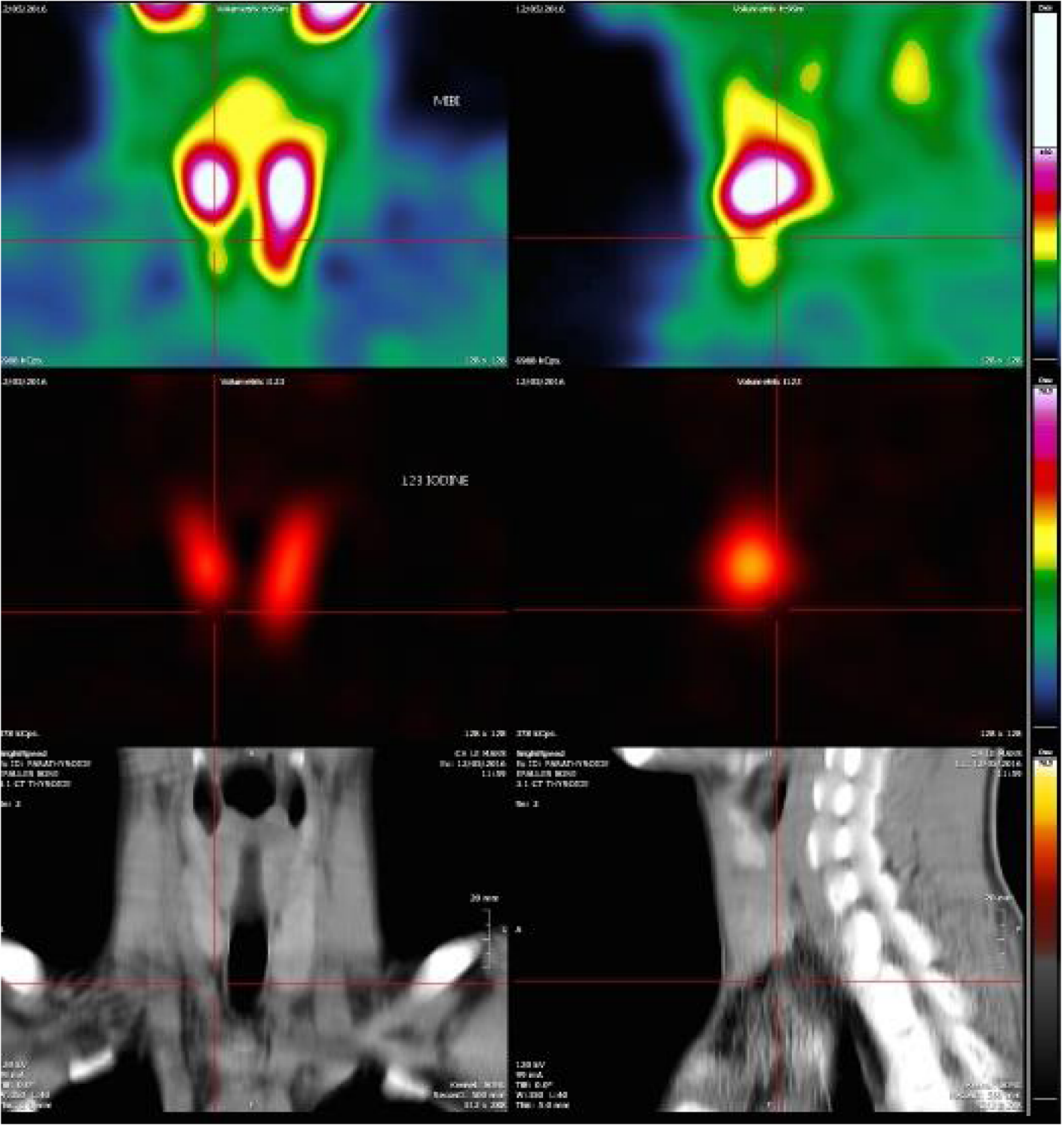
Parathyroid scintigraphy: substraction images are compatible with the presence of bilateral inferior parathyroid adenomas

The patient’s parathyroid hormone levels remained high (> 1500 ng/l) and he developed hypercalcemia after the start of chronic dialysis. These alterations did not respond to dialysis treatment (Kt/V, according to the Daugirdas 2 formula was always over 1.7); he did not take Cinacalcet, as prescribed, and his PTH decreased only partially during dialysis, in spite of hypercalcemia at the end of treatment (1604 ng/l before to 1087 ng/l after dialysis, in spite of a total calcium increase from 2.49 mmol/l to 2.90 mmol/l at the end of dialysis), suggesting secondary-tertiary hyperparathyroidism. Parathyroid scintigraphy was compatible with the presence of bilateral inferior parathyroid adenomas (Fig. 1).

As optimal dialysis had failed to produce improvement, in view of kidney transplantation, our patient underwent subtotal parathyroidectomy 1 year after dialysis start. The histologic analysis of the glands confirmed the presence of diffuse parenchyma hyperplasia with cellular hypertrophy, clear organoid architecture (pseudoglandular structures) and small oxyphilic cells, with no atypia or mitosis. His calcium and PTH values dropped (induction PTH = 1317 ng/l; PTH after PTX = 161 ng/l), and he developed a symptomatic hungry bone syndrome (Table 1). After PTX, his blood pressure stabilized in the 100–140/50–80 range (min and max values in the second month after parathyroidectomy), and has remained normal without treatment up to the time of the present report (5 months later).

## Systematic review of the literature

### Methods

The PICO criteria were adopted as follows: Patients: people with secondary or tertiary hyperparathyroidism in dialysis; Intervention: parathyroidectomy; Comparators: the same patients before and after the intervention; Outcome: changes in blood pressure.

The eligibility criteria were broad, because we expected retrieval of a low number of papers with heterogeneous designs and definitions. Consequently, we included all published studies that dealt with blood pressure following parathyroidectomy. We included all study designs, i.e. prospective or retrospective cohort studies, case-control studies, trial-based analyses, and case reports. Reviews, whether or not systematic, were also searched to check for papers that had escaped from our wide search strategy.

**Fig. 2:**
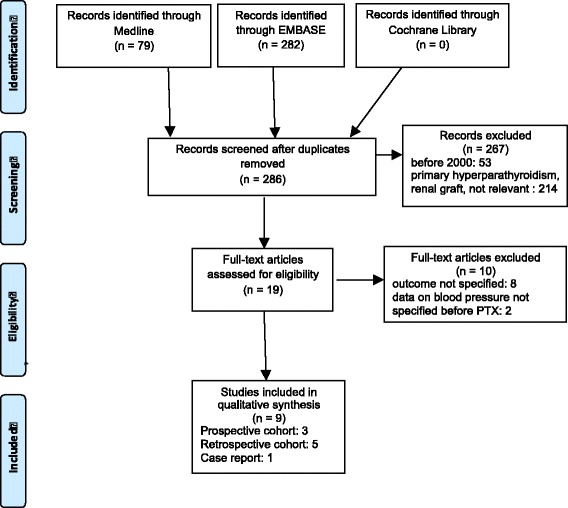
Flow chart of paper selection for the systematic review.Note: examples of titles of non relevant papers: “The Use of Calcimimetics for the Treatment of Secondary Hyperparathyroidism: A 10 Year Evidence Review.” or “Calciphylaxis: a case report”

We searched PubMed indexed for MEDLINE, Embase and the Cochrane Review database from January 1, 2000 to December 31, 2016 using a combination of MeSH terms and keywords related to hyperparathyroidism, hypertension and dialysis, including: hyperparathyroidism, parathyroidectomy; dialysis, hemodialysis, hemofiltration, hemodiafiltration, renal replacement therapy; blood pressure, hypertension, hypotension, hypertensive and hypotensive. We also checked the reference lists of relevant articles and of studies (Fig. 2). Although we did not limit our search to by language, we only considered studies published after 2000, to enable us to focus on the considerable changes in the management of the hyperparathyroidism that have occurred in the new millennium, and thereby contextualize our clinical decision in keeping with the current recommendations on hyperparathyroidism in dialysis patients.

Articles identified were screened for eligibility solely on the basis of the content: papers reporting on any outcome regarding blood pressure after parathyroidectomy were selected. Data extraction was performed by AS and double checked by GBP; discrepancies were resolved through discussion.

Statistical analysis (meta-analysis) was performed using Metanalyst Beta 3.13, for the outcome blood pressure.

### Results

The search strategy identified 286 articles, of which 9 met the inclusion criteria: 1 case report, 5 retrospective studies and 3 prospective studies, dealing with a total of 187 patients (Fig. 2). The main characteristics of the studies are reported in Tables 2 and 3.

**Table 2:** The main aim and study design of the papers reviewed

Ref	Author, year	Country	Aim	Type of study
6	Lin, 2013	Taiwan	To determine the association of PTX with major cardiovascular events in nondiabetic patients with severe secondary hyperparathyroidism: comparison between medical and surgical treatments	Retrospective
7	Shih, 2013	Taiwan	To analyze the frequency of intradialytic hypotension and cardiovascular function before and after PTX	Retrospective
8	Leiba, 2013	Israel	To describe severe long-lasting hypotension starting immediately after parathyroidectomy (3 cases)	Case report
9	De Santo, 2010	Italy	To investigate the prevalence of alexithymia, sleep disorders and depression in patients on hemodialysis refractory hyperparathyroidism needing PTX; to study the effects of PTX on alexithymia	Prospective
10	Esposito, 2008	Italy	To study the effects of PTX on the sleep disorders of insomniacs on maintenance hemodialysis	Prospective
11	Chow, 2003	China	To study changes in health-related quality of life, pruritus and left ventricular hypertrophy in dialysis patients with tertiary hyperparathyroidism before and after PTX	Prospective
12	Almirall, 2002*	Spain	To investigate the relationship between hyperparathyroidism and hypertension	Retrospective
13	Saatci, 2002	Turkey	To investigate the effects of medical and surgical therapy of hyperparathyroidism on blood pressure and lipid levels in chronic renal failure	Retrospective
14	Coen, 2001	Italy	To evaluate the long-term results of PTX on BP and anemia	Retrospective

*Letter to the editor

**Table 3:** Clinical and laboratory data before and after PTX

Ref	Author, year	N cases	Age (years)	PTH pre/post (pg/ml)	BP pre/post (mmHg)	Other	Antihypertensive treatment
6	Lin, 2013	30*	53.3 ± 13.3	1012 ± 247 vs 136 ± 157 (*P* < 0.001)	Sys:148 ± 17 vs 139 ± 18 Dia: 79 ± 10 vs 74 ± 12 (*P* < 0.009; < 0.007)	Ca, PO4, Ca x Ph, hemoglobin improved after PTX;	No data
7	Shih, 2013	21	57.4 ± 12	1011(611–1402) vs 19.1 (10.3–61.6) 6 mos (*P* < 0.001)	Sys:138.9 ± 16.4 vs 121.2 ± 18.6 Dia: 77.6 ± 8.3 vs 69.2 ± 9.8 (*P* < 0.001)	Improved heart function; reduced intradialytic hypotension.	Pre PTX: 1.14 ± 1.11 drugs Post PTX: 0.67 ± 1.2 drugs
8	Leiba, 2013	3	45, 46, 30	2408 / 6 7000 / 2000 3642 / 16–30	Sys: 140; 180–200; 175–210 vs 60; 50; 100 Dia: 90; 100; 120 vs 40; 30; 60		Pre PTX Case 1: no data Case 2: clonidine, amlodipine, atenolol doxazosin Case 3: clonidine, minoxidil
9	De Santo, 2010	40	55.8 ± 6.8	1.299.6 / 46.8	Sys: 139 vs 135 Dia: 83 vs 81 (*P* < 0.001, *P* = 0.001)		treated Pre PTX 100% Post PTX 72.5%
10	Esposito, 2008	16	54.6 ± 13	1434 ± 400 vs 40.3 ± 37.4	Sys: 138.9 ± 18.39 vs 130.8 ± 23.47 (*P* = 0.05) Dia: 83.1 ± 11.84 vs 78.8 ± 6.7 (*P* = 0.05)	Beneficial effects of PTX on sleep disorders.	Pre PTX, all on combined treatment** Post PTX: 12 /16 on antihypertensive drugs.
11	Chow, 2003	12	50 ± 11	243.6 ± 22.6 vs 9.2 ± 13.4	Sys: 141 ± 16 vs 145 ± 16 Dia: 77 ± 10 vs 80 ± 10 *P* = NS	No effect on pruritus; regression of left ventricular hypertrophy	Pre PTX: ACEi: 2 (17%); Post PTX: ACEi 1 (8%) *P* = NS
12	Almirall, 2002	17	NS	1056 ± 803 (pre-PTX)	Sys: 140.5 ± 17.3 vs 141 ± 20.8 Dia: 81.6 ± 9.3 vs 80.4 ± 9.3 *P* = NS	No effect on heart rate, dry body weight	No difference after PTX
13	Saatci, 2002	13	41.38 ± 10.85	1210.25 ± 262.2 vs 172.88 ± 155.88	Sys: 126 ± 25.9 vs 102.8 ± 20.5 (*P* < 0.01) Dia: 74 ± 16.46 vs 64.44 ± 15.89 (*P* < 0.04)	Decline in BP, if PTH < 200 ng/l. Benefits to triglyceride levels	Groups similar at baseline
14	Coen G, 2001	45	56 ± 11	1313.4 ± 1.004.1 vs 214.2 ± 357.4 (1 week-6 months)	Sys: 134.7 ± 29.2 vs 125.9 ± 25.8 Dia: 81.6 ± 14.6 vs 77.8 ± 12.9 (*P* < 0.001)	No difference between subtotal or total PTX; increased erythropoietin level	Anti-hypertensive number and doses reduced post PTX

* 23 controls; ** combined treatment: (betablockers, calcium channel blockers, ACEi, ARB receptors antagonists); BP = blood pressure; sys = systolic blood pressure; dia = diastolic blood pressure; PTX = parathyroidectomy

The papers were in general homogeneous for age of patients (mean of about 55 years, with the exception of the previously mentioned case report in which patients were younger), but non-homogenous for the setting of the study (five from Asia and four from Europe) for indications for parathyroidectomy, and selection of patients. For example, in the study by Lin, PTX was recommended for severe secondary hyperparathyroidism defined as PTH level higher than 800 pg/ml associated with bone or joint pain, muscle weakness, irritability, itching, bone loss, anemia resistant to erythropoietin, cardiomyopathy or calciphylaxis [6]. Instead in Saatci’s study, patients with PTH above 200 ng/L, who were unresponsive to vitamin D for 6 months, underwent total PTX with autotransplantation [13]. In the study by Coen et al., PTX was performed for intractable pruritus, persistent hypercalcemia and in one case for generalized bone pain [14].

Overall, within the baseline heterogeneity, a trend towards an improvement in BP control was observed in most case series: 6/8 papers (Table 3). Furthermore, in the case report a sharp reduction in blood pressure was recorded in the three cases described [8].

**Fig. 3:**
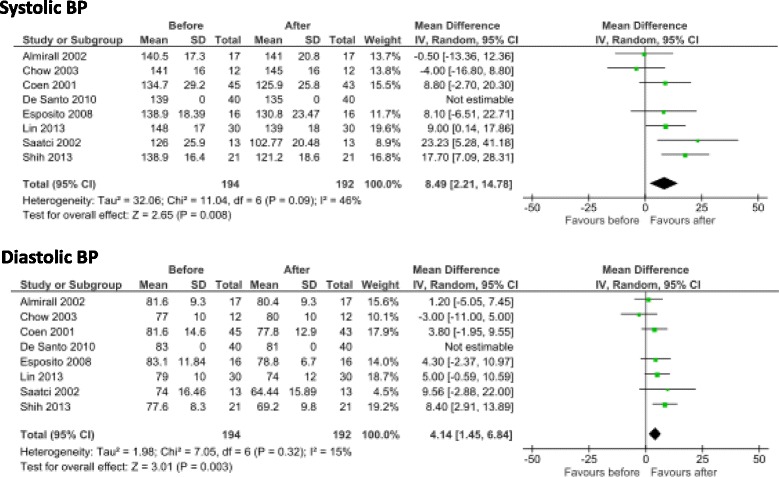
Metaanalysis of the selected studies

The meta-analysis showed a significant, albeit moderate improvement. in particular in systolic BP (difference: systolic BP 8.49 (CI 2.21–14.58) mmHg; diastolic BP decrease, 4.14 (CI 1.45–6.84) mmHg was significant albeit of a lesser degree (Fig. 3).

Our evaluation of the effect on blood pressure was limited by the lack of clear information on anti-hypertensive agents, which have probably been reduced in a relevant number of cases, as judged from the comments in the papers by Shih, Esposito and Chow (Table 3).

## Discussion

The case described has features that make it an interesting example of the complex relationship between severe hyperparathyroidism and hypertension.

The patient is young and has a non-immunologic disease. His compliance with drug therapy was poor, while his compliance with dialysis treatment was excellent. Tests for the cause of a so far uncharacterized interstitial disease identified no immunologic interference. His lack of compliance with chronic treatments allowed us to observe the effect of the metabolic derangements and of their correction on his blood pressure pattern without the interference of pharmacologic treatment and his good compliance with dialysis enabled us to analyze the effect of PTX without the interference of a low dialysis dose.

The prompt, complete and long-lasting normalization of blood pressure was unexpected, and this led us to review the current literature, so that we could contextualize our case and reflect on whether and how this positive effect of treatment could be expected in similar situations.

The relationship between hyperparathyroidism and hypertension in dialysis patients was reported in the 1980s and 1990s, albeit with conflicting results: Ifudu showed that in hypertensive dialysis patients PTX failed to correct hypertension, suggesting a minor effect of PTH in dialysis hypertension at least in relation to volume overload [16]. Zucchelli obtained similar results, inferring that very high plasma PTH was not associated with cardiovascular impairment [17]. Instead, although Goldsmith did not find a clear relationship between a PTH decrease after PTX and any immediate measure of BP, the study provided evidence for a long-term antihypertensive effect, progressively developing over 3–6 months [20]. The authors hypothesized a “resetting” of intracellular calcium, or slow removal of calcium from the blood vessel walls. Along similar lines, Pizzarelli proposed that a BP decrease post-PTX is related to calcium efflux from the vessel walls [15].

Recent data confirm the pathogenic effect of hyperparathyroidism, elucidating the molecular effects and clearly indicating that vascular stiffness is not only mediated by calcium and phosphate accumulation, but also by PTH itself, via PTH2 receptors [25–27]. Elevated PTH levels can increase production of collagen by vascular smooth muscle cells and directly affect cardiac function, reducing contractility and inducing ventricular hypertrophy [28, 29]. These changes are at least partially reversible, since PTX improves arterial stiffness, and has been associated with longer patient survival, provided that calcium concentrations are normalised [22, 23, 25–30].

Unlike the elegant pathophysiologic studies, the recent data on blood pressure before and after PTX, which we gathered for our systematic review and meta-analysis, are highly heterogeneous and their interpretation is biased by a lack of precise information on concomitant drug therapy.

However, the limited information available in most of the papers retrieved suggests that the positive effect on BP was recorded in the setting of equal or reduced anti-hypertensive doses, thus further underlining the clinical relevance of BP decrease after PTX [8–13, 15].

In this context, within all the limits mentioned above, our meta-analysis allowed us to quantify the BP difference, which was higher for systolic BP (8.49 (CI 2.21–14.58) mmHg), in keeping with a reduction in vascular stiffness, and was significant, but of a lesser entity, for diastolic BP (4.14 (CI 1.45–6.84) mmHg).

It is noteworthy that, in the only case report we were able to retrieve, the differences were more important and were abrupt, and the three cases described suggest that patients may actually shift from severe hypertension to symptomatic hypotension after PTX [8]. A similar, but less extreme pattern was observed in our case, in which the patient’s young age and short dialysis follow-up may have made possible preservation of vascular reactivity. In fact, the outcome was fully positive, and the patient was simultaneously cured of two severe cardiovascular risk factors (hyperparathyroidism and hypertension). In keeping with the series mentioned above, our case suggests considering the possibility that PTX may rapidly reset BP control, at least in some patients (Table 1) [9].

## Conclusion

Our review of the literature, within the limit of heterogeneity and of limited quality, suggests that PTX exerts a beneficial effect on blood pressure, leading to a significant decrease in both systolic and diastolic BP; the higher effect on systolic BP suggests a beneficial reduction of vascular stiffness. The results are however biased by the lack of information on drug therapy, an issue that should be addressed in future prospective studies.

Our case, like the ones of three other patients reported in the literature, serves as a reminder that the effect on blood pressure is highly unpredictable. Full correction may occur, as it did in our patient, but even symptomatic hypotension sometimes, albeit rarely, has been found in patients after PTX. Further prospective studies are needed that better quantify the entity of the changes, the risk of extreme BP reduction and interference with pharmacologic treatment, to enable us to make use of these findings in our clinical judgments and counseling.
